# Tensile Characterization of Single-Walled Carbon Nanotubes with Helical Structural Defects

**DOI:** 10.1038/srep20324

**Published:** 2016-02-04

**Authors:** Young I. Jhon, Chulki Kim, Minah Seo, Woon Jo Cho, Seok Lee, Young Min Jhon

**Affiliations:** 1 Sensor System Research Center, Korea Institute of Science and Technology, Seoul 136-791, Korea; 2 Center for Opto-Electronic Conversion System, Korea Institute of Science and Technology, Seoul 136-791, Korea

## Abstract

Recently, evidence was presented that certain single-walled carbon nanotubes (SWNTs) possess helical defective traces, exhibiting distinct cleaved lines, yet their mechanical characterization remains a challenge. On the basis of the spiral growth model of SWNTs, here we present atomic details of helical defects and investigate how the tensile behaviors of SWNTs change with their presence using molecular dynamics simulations. SWNTs have exhibited substantially lower tensile strength and strain than theoretical results obtained from a seamless tubular structure, whose physical origin cannot be explained either by any known SWNT defects so far. We find that this long-lasting puzzle could be explained by assuming helical defects in SWNTs, exhibiting excellent agreement with experimental observation. The mechanism of this tensile process is elucidated by analyzing atomic stress distribution and evolution, and the effects of the chirality and diameter of SWNTs on this phenomenon are examined based on linear elastic fracture mechanics. This work contributes significantly to our understanding of the growth mechanism, defect hierarchies, and mechanical properties of SWNTs.

Carbon nanotubes have gained a great amount of attention since their discovery^1^ due to their superb mechanical and electrical properties^2^,^3^,^4^ as well as excellent compatibility with polymers and biological objects such as polypropylene and human mesenchymal stem cells^5^,^6^,^7^. In particular, tremendous efforts have been placed to characterize the structure and properties of single-walled carbon nanotubes (SWNTs)^8^,^9^,^10^,^11^,^12^,^13^,^14^ in which most of theoretical studies have been performed by assuming a seamless tubular structure or by introducing several well-known point defects in the structure^15^,^16^,^17^,^18^.

However, throughout the long research history of SWNTs, a remarkable discrepancy has been consistently observed between experimental and theoretical results of the tensile strength and yield strain of SWNTs, which raises fundamental questions about their origin^19^,^20^,^21^,^22^,^23^. (It should be noted that this is true for SWNTs, not multi-walled carbon nanotubes.) That is, experiments have shown that the tensile strength and yield strain of SWNTs is of 30–45 GPa and 5.3–5.8%, respectively, exhibiting a three- to four-fold deviation from the theoretical results obtained assuming seamless tubular SWNTs. Two potential reasons have mainly been discussed to explain these unexpected experimental results. The first issue concerns the weak anchoring of SWNTs to tensile devices. However, SWNTs are proven to be robust in maintaining their end-point connections even after tensile failure (they can be broken over the repeated measurements, but we only consider the cases of robust end-point connections in evaluating the tensile strength), which indicates that the first issue is not a critical reason^19^. This was further confirmed by the fact that SWNTs with different levels of end-point adhesion exhibit almost the same maximum tensile strain (~5%) as each other’s; SWNTs lie on a trenched substrate in one case^23^, forming a large contact area with the substrate, while SWNTs are anchored to an AFM tip in the other case^19^. The second is associated with mechanical deterioration caused by Stone-Thrower-Wales (STW) defects or vacancy defects created in SWNTs during the synthetic process. However, theoretical studies have shown that tensile strength degradation caused by any of known defects is much less severe than what has been experimentally observed^24^,^25^. Using molecular dynamic simulations, Zhang *et al.* found that rather large holes or slits must exist in SWNTs to obtain results that are equivalent to the experimental data^25^. However, they were not able to find appropriate sources to generate such defects in SWNTs at that time.

Meanwhile, despite enormous research that have been performed on SWNTs, the growth mechanism of SWNTs has remained unknown for decades until a screw-dislocation-like (SDL) growth model was proposed by Ding *et al.* in 2009^26^. Soon after, this model was verified through the experimental observation of a distinct spiral growth of SWNTs via field emission microscopy^27^. In conjunction with this model, it is worth noting that recently a pioneering work is reported by Lee *et al.* in which residual traces of the spiral growth and its associated helical defects are discovered in SWNTs using high-resolution transmission electron microscopy (HRTEM) and atomic force microscopy (AFM)^22^,^28^. They inferred that these helical defects could be the key to the puzzling tensile problem of SWNTs mentioned above.

In this work, we have validated this intriguing hypothesis by first presenting atomic details of helical defects based on a SDL growth model, and then by investigating how the tensile properties of SWNTs change in the presence of these defects using molecular dynamics simulations. We also find that even a small quantity of helical defects in SWNTs can lead to the tensile strength degradation equivalent with experimental results, whereas STW defects or vacancy defects in heavy density cannot reproduce experimental observation, which supports the tensile fracture mechanism described above more firmly. All the computations are performed using LAMMPS software package^29^ with AIREBO potential^30^ that has been widely used to study the mechanical properties of various carbon nanomaterials such as carbon nanotubes, fullerenes, and graphenes^31^,^32^,^33^,^34^,^35^.

## Results

### Atomistic model of helical defect morphology

**Figure 1:**
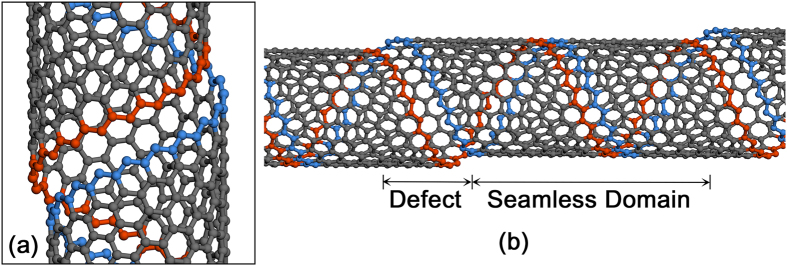
Initial structures for the generation of helical defects in SWNTs. (**a**) The structure representing lattice mismatches (the smallest atomic mismatches) during the spiral growth of SWNTs and (**b**) The overall view of SWNTs containing these lattice matches. Blue and red indicate the opposite edges of a graphene nanoribbon.

**Figure 2:**
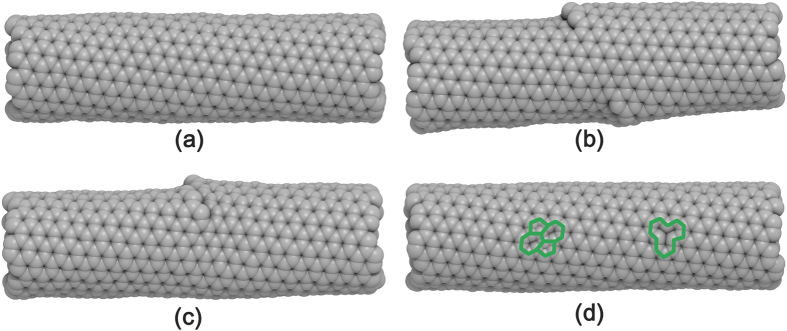
Various possible structures of SWNTs. Structures of (**a**) SWNT-PR, (**b**) SWNT-HL1, (**c**) SWNT-HL2, and (**d**) SWNT-STW/SWNT-MV in which STW (left) and MV defects (right) are shown in green in a single SWNT for simplicity.

We assume that SWNTs are formed through the spiral growth of the “zigzag-edged” graphene nanoribbons as suggested in Ding *et al.*’s SDL growth model^26^. Thereby, by adopting a zigzag-edged graphene helix as the structural basis of the SWNTs, we hypothesize that the graphene helix is perfectly zipped in many turns, but the turn occasionally mismatches (Fig. 1) since the front growth of the graphene nanoribbon of the helix is quite fast as observed in the experiments^27^. In this model, the axial distance between the defective regions is determined by the number of turns in which the graphene helix is completely matched, and thus helical defects can be extremely localized, allowing for a large portion of the SWNT domains to be seamlessly tubular (Fig. 1b) being compatible with normal TEM images of SWNTs. We infer that the width of the graphene nanoribbon is commensurate with the magnitude of the Burgers vector defined in the SDL growth model^26^,^36^. It is worth noting that the synthesis of SWNTs via twisted (*i.e.*, not simply rolled up, but helically coiled) nanoribbons has actually been realized in experiments and in simulations^37^,^38^, supporting the above model firmly. Starting from SWNT structures with slightly mismatched helical turns (Fig. 1), we obtain the equilibrated structures via geometric optimization followed by NPT and NVT simulations (see the Method section for computation details). Depending on the subtle differences in the system conditions, three different, thermodynamically stable SWNTs are obtained consistently as shown in Fig. 2. The first is made by zipping up the cracks, and hence restoring it back into a pristine SWNT (Fig. 2a). The second is a SWNT with topologically-staggered nodelike helical defects (Fig. 2b and S1a), and the third is a SWNT with topologically-coherent nodelike helical defects (Fig. 2c and S1b). These three types of SWNTs are denoted hereafter as SWNT-PR, SWNT-HL1, and SWNT-HL2, respectively. Interestingly, the SWNT-HL1 and SWNT-HL2 resemble several anomalous TEM images of SWNTs previously reported^20^,^21^, as indicated by Lee *et al.*^22^.

### Tensile properties of SWNTs with helical defects

To investigate how these helical defects affect the tensile properties of the SWNTs, we carry out a series of tensile molecular dynamics simulations for the SWNT-PR, SWNT-HL1, and SWNT-HL2 configurations. The chiralities of all these SWNTs are set to (12, 8) since (12, 8) SWNTs have almost the same diameter (~1.4 nm) as the one (~1.36 nm) in Yu *et al.*’s experiment^19^, and they also serve as a good SWNT representative since the SWNT population increases with the sine of the chiral angle 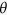 (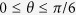)^26^ that is defined as follows:


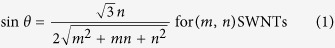


**Figure 3:**
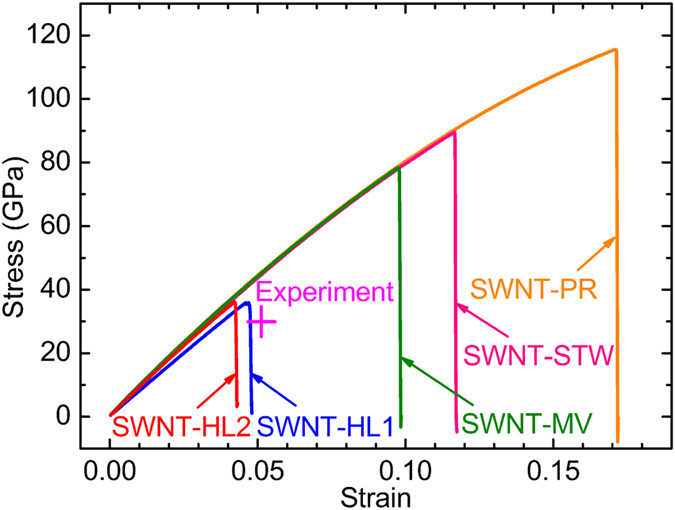
Tensile stress-strain curves of SWNTs with various types of structural defects. The experimental tensile failure point is marked by a magenta cross.

The tensile strength and yield strain of SWNT-PR are calculated to be ~117.1 GPa and ~17.6%, respectively, showing good agreement with the previous results obtained from pristine SWNTs^39^. In contrast to this, the computation indicates that the tensile strength and yield strain of SWNT-HL1 are ~35.9 GPa and ~4.7%, respectively, and those of SWNT-HL2 are ~35.9 GPa and ~4.2%. These tensile strength values of SWNT-HL1 and SWNT-HL2 are both significantly smaller than that (~117.1 GPa) of SWNT-PR (i.e., a pristine SWNT). However, they are in excellent agreement with the experimental values obtained by Yu *et al.* (30 GPa) and Walters *et al.* (45 GPa, which was indirectly obtained by multiplying the measured maximum tensile strain by an assumed elastic constant of 1.5 TPa)^19^,^23^ as shown Fig. 3 and Table 1. The yield strains of SWNT-HL1 (4.7%) and SWNT-HL2 (4.2%) are also in good accordance with the experimental values of Yu *et al.* (5.3%) and Walters *et al.* (5.8%), whereas the yield strain of SWNT-PR (17.6%) is significantly greater than experiment observation.

In order to show clearly that any of the known defects in SWNTs cannot be responsible for experimentally-observed, anomalously-low tensile strength and strain of (high-quality arc-discharge) SWNTs, we have also performed tensile molecular dynamics simulations of SWNTs in the presence of monovacancy (MV) and STW defects (the corresponding SWNTs are denoted hereafter as SWNT-MV and SWNT-STW, respectively). The tensile strength and yield strain of SWNT-MV are estimated to be ~79.9 GPa and ~10.0%, respectively, while those of the SWNT-STW are ~90.2 GPa and ~11.8%, respectively.

These results agree well with the previous studies (Table 1) in which the tensile strength of the (5, 5) SWNTs with STW defects decreases by 18–25% relative to that of pristine SWNTs^39^, while the tensile strength of the (5, 5) SWNTs with mono- or di-vacancy (DV) defects decreases by 20–32%^24^. Manifestly, all of these tensile strength values are far beyond the experimental values (Fig. 3 and Table 1), even considering the experimental uncertainties^19^,^23^.

### Defect-density dependence of tensile properties of SWNTs

**Figure 4:**
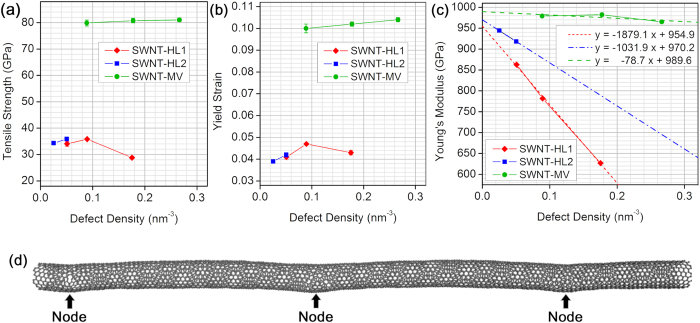
The effect of defect densities on the tensile properties of SWNTs. (**a**) Tensile strength, (**b**) yield strain, and (**c**) Young’s modulus of SWNT-HL1, SWNT-HL2, and SWNT-MV, plotted as a function of the density of nodelike helical defects and MV defects. (**d**) The structure of SWNT-HL1 with the nodelike helical defect density of 0.051 nm^−1^.

Tensile failure occurs at the weakest point of SWNTs. Therefore, even in the case where helical defects are created at a very low level, their presence should be fatal to the tensile stability of SWNTs. In other words, seemingly perfect SWNTs may possess helical defects and could lead to a remarkably degraded tensile strength, as observed in the experiments^19^,^23^, which might be more common than is expected. To address this issue at a deeper level, we further examine how the tensile behaviors of the SWNT-HL1 and SWNT-HL2 change as the density of nodelike helical defects varies. We find that the tensile strength of SWNTs remains almost constant for the variation of the helical defect density (Fig. 4a), except for the case with the extremely high density of helical defects (the tensile strength of SWNT-HL1 decreases from 34.1 to 28.8 GPa as the helical defect density increases from 0.05 to 0.175 nm^−1^).

Negligible defect-density dependence of the tensile strength of SWNTs has also been observed in the case of the MV defects (Fig. 4a). We find that the same trends are applicable to the yield strains as well (Fig. 4b). These results indicate that we cannot explain the anomalously low tensile strength of SWNTs as a result of the large quantity of STW and MV defects in the SWNTs. We therefore claim that it is not the defect density but the defect type that determines the tensile strength of SWNTs.

In contrast to the tensile strength and the yield strain, the Young’s modulus (YM) of SWNTs rather distinctly decreases as the nodal density increases (Fig. 4c), although it remains almost constant in the case of the MV defects. The large reduction in YM observed in SWNT-HL1 and SWNT-HL2 is attributed to substantially broken structural moieties present in the helical defects. The degree of reduction in the YM is drastically weakened as the helical density decreases. A linear fitting of these YM data of SWNT-HL1 and SWNT-HL2 indicates that as the helical defect density decreases, the YM approaches that (~966.2 GPa) of pristine SWNTs (Fig. 4c). The slope of the fitting line for SWNT-HL1 is about twice as large as that in SWNT-HL2, presumably due to the fact that SWNT-HL1 has two broken spots per a node while SWNT-HL2 has a single broken spot. The structure of SWNT-HL1 with the helical defect density of 0.051 nm^−1^ is illustrated in Fig. 4d.

### Atomic-scale Understanding of Tensile Fracture Mechanism

**Figure 5:**
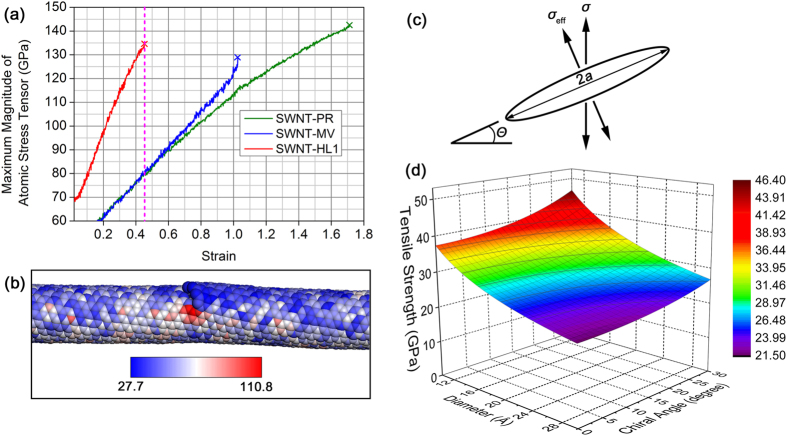
A crack-based mechanism for the tensile fracture of SWNTs with helical defects. (**a**) Evolution of the maximum magnitude of atomic stress tensors in SWNT-PR, SWNT-HL1, and SWNT-MV under tensile loading. The short-dashed magenta line indicates the yield strain of SWNT-HL1. (**b**) Atomically-resolved stress distribution developed in SWNT-HL1 just before tensile failure. (**c**) Schematic of a crack (the length is 2*a*) pertaining to the helical defects of SWNTs (the chiral angle is 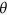) under tensile loading. (**d**) The tensile strength of SWNTs containing helical defects plotted as a function of the chiral angle and the diameter in the regime of LEFM.

To understand the tensile fracture mechanism of the above phenomenon, we investigate the atomically-resolved stress evolution during the tensile processes of SWNT-PR, SWNT-HL1, and SWNT-MV. We find that the maximum magnitudes of the atomic stress tensors in these SWNTs are quite similar to each other at tensile failure, ranging from 130–140 GPa (Fig. 5a). It is worth noting that these values multiplied by a carbon atomic volume of SWNTs (153.18–164.96 kcal/mol) are on the same order of magnitude as the carbon-carbon double bond energy (146 kcal/mol). We find that the stress concentration factor of the helical defects is about 1.7 times as large as that of MVs or pristine SWNT structures at a yield strain of SWNT-HL1 (4.52%), as indicated by the short-dashed magenta line in Fig. 5a. The spatial atomic stress distribution of the SWNT-HL1 obtained at tensile failure is shown in Fig. 5b, indicating that the stress is highly concentrated at the tip of the cracks in the helical defects.

Finally, we investigate the chirality and diameter effects of the SWNTs on their helical-defect-induced tensile strength degradation by using the linear elastic fracture mechanics (LEFM)^40^. In this mechanical regime, whether a crack heals or propagates is determined by the stress intensity factor *K*_I_ that is introduced by Irwin’s modification of the Griffith theory^41^. By applying the LEFM to our system, we obtain the following relation:


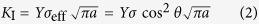


where *Y* is a constant that depends on the crack opening mode and the geometry of the specimen, 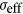 is the effective stress normal to the crack, 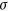 is the tensile stress applied to the SWNTs, 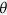 is the chiral angle (see Eq. (1)), and *a* is half of the crack length (Fig. 5c). Assuming that the value of *K*_I_/*Y* is constant and that the crack length of the ND defects is proportional to the diameter of the SWNTs divided by the cosine of the chiral angle (see Figure S2 for detailed explanation), the tensile strength of the SWNTs is plotted as a function of their chiral angle and diameter (Fig. 5d). The results show that the tensile strength can vary between 20.6 and 47.7 GPa for a change in diameter between 10 and 30 Å and the full chiral angle change of 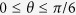, which is still reasonable when compared to the experimental results. Actually, the lower tensile strength values of this estimated range will seldom be attained since the diameter of SWNTs is generally of 10–20 Å, and SWNTs are formed proportionally to the chiral angle.

## Discussion

Apart from Lee *et al.*’s experimental evidence for helical defective traces in SWNTs, it is reasonable to imagine that certain lattice mismatches may occur during the spiral growth of SWNTs, which deserves to be investigated theoretically. Arc-discharge methods yield the most high-quality products of SWNTs. However, tensile experiments using these products have consistently shown considerably lower tensile strength and strain than theoretical prediction so far, even considering possible STW and vacancy defects and experimental uncertainty. It is a surprise that this long-lasting puzzle can be explained clearly by admitting the presence of helical defects in SWNTs, exhibiting excellent agreement with experimental observation. We find that the tensile strength of SWNTs is determined not by the density of defects, but by the type of defects solely. This fact indicates that even a very rare occurrence of lattice mismatches during the spiral growth of SWNTs can lead to the same amount of tensile degradation as in the cases of their heavy density, supporting our tensile fracture model firmly. We infer that these lattice mismatches may also be related with a heterojunction between SWNTs with different chiralities since it naturally allows a change in the chirality of SWNTs in the regime of the spiral growth, which will be our next research topic.

In our analysis, a tensile failure point is determined to be the point that exhibits the maximum tensile stress followed by its prestigious drop (Figure S3a). In fact, considerable residual stresses occur for a while (Figure S3b) even after tensile failure of SWNT-ND1 due to the connection via graphene nanoribbons that come from unravelling of nodal structures (Figure S4). Such residual mechanical response could be experimentally observed in nm-scale samples like our simulation systems. However it is hardly detected in μm-scale samples, which are on the same order of magnitude as system dimensions of most experiments^19^,^23^, because such inhomogeneous deformation appears very shortly in μm-scale samples due to the extremely large ratio (10^3^–10^4^) of a sample length to a nodal region length. However, we infer that the trace of graphene nanoribbons can be captured at the tensile fracture sections in μm-scale SWNT samples, and suggest experimentalists to carefully probe this point which may distinctly differ from that expected from pristine SWNTs. We believe that this work contributes greatly not only to our understanding of a growth mechanism, defect hierarchies, and mechanical properties of SWNTs, but also to potential applications of their defect-based engineering in the future.

## Methods

### Equilibrated structures

Molecular dynamics simulations are performed with a time step of 0.5 fs using the LAMMPS (Large-scale Atomic/Molecular Massively Parallel Simulator) software package^29^. The simulation systems are constructed to meet a periodic boundary condition. For interactions between carbon atoms, we employed AIREBO (adaptive intermolecular reactive empirical bond order) potential^30^. The cut-off radius of the interatomic potential was set to be 2.0 Å to avoid spuriously high bond forces and unphysical results near the fracture region. The dimensions of the simulation system and atomic coordinates are first optimized using a gradient-based minimization method with tolerance criteria of 10^−8^ eV/Å in force and/or 10^−8^ eV in energy. NPT ensemble simulation is subsequently performed for 7 × 10^5^ steps at 1 atm and 300 K and the system is further equilibrated using NVT ensemble simulation for 3 × 10^5^ steps.

### Tensile processes

The system is elongated with a strain rate of 0.1 ns^−1^ in the specific tensile direction where a non-equilibrium molecular dynamics simulation is employed to describe the non-thermal streaming velocities of the continuously strained system using the SLLOD equations of motion coupled to a Nose-Hoover thermostat^42^. All the tensile simulations have been performed three times to avoid a possible statistical error. SLLOD algorithm is given as follows:


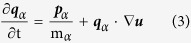



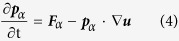


where 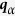, 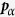, and 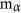 are a position, momentum, and mass of 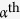
atom, respectively, *t* is the time, and 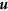 is the streaming velocity. Meanwhile, the atomic stress tensor of individual carbon atom is calculated by:


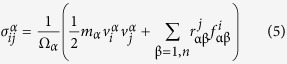


where, i and j are components in Cartesian coordinates, 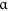 and 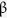 are the atomic indices, 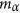 and 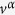 are the mass and velocity of 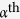
atom, 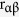 and 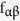 are the distance and force between 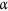 and 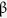 atoms, and 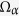 is the volume of 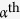 atom.

## Additional Information

**How to cite this article**: Jhon, Y. I. *et al.* Tensile Characterization of Single-Walled Carbon Nanotubes with Helical Structural Defects. *Sci. Rep.* **6**, 20324; doi: 10.1038/srep20324 (2016).

## Supplementary Material

Supplementary Information

## Figures and Tables

**Table 1:** Tensile strength, yield strain, and Young’s modulus of SWNTs obtained from various theoretical models and experiments.

	Stress (GPa)	Strain	YM (GPa)
SWNT (12, 8)†	117.098 ± 1.377	0.176 ± 0.004	966.246 ± 4.736
SWNT (9, 0)^39^	94.0	0.164	939.1
SWNT (5, 5)^39^	123.0	0.216	894.7
SWNT (12, 8) with MV†	79.885 ± 1.129	0.100 ± 0.002	979.244 ± 3.821
SWNT (5, 5) with DV^25^	71.3	0.117	n/a
SWNT (12, 8) with STW†	90.150 ± 1.445	0.118 ± 0.003	982.737 ± 7.007
SWNT (5, 5) with STW^24^	92.44	0.115	n/a
SWNT (12, 8) with HL1†	35.860 ± 0.145	0.047 ± 0.000	781.835 ± 5.022
SWNT (12, 8) with HL2^†^	35.881 ± 0.664	0.042 ± 0.001	918.197 ± 8.442
SWNT (experiment)^19^	13–52 (mean:30)	<0.053	320–1470 (mean:1006)
SWNT (experiment)^23^	45	<0.058	n/a

^†^This work.
