# Supplementary material for: Tensile Characterization of Single-Walled Carbon Nanotubes with Helical Structural Defects
Source: Sci Rep. 2016 Feb 4;6:20324. doi: 10.1038/srep20324 (PMC4740892; doi:10.1038/srep20324)
Supplement: Supplementary Information [file srep20324-s1.pdf]

Supplementary Material for

Tensile Characterization of Single-Walled Carbon Nanotubes with Helical Structural Defects

Young In Jhon,[1] Chulki Kim,[1] Minah Seo,[1] Woon Jo Cho,[2] Seok Lee,[1] and Young Min Jhon[1,*]

[1]Sensor System Research Center, Korea Institute of Science and Technology, Seoul 136-791, Korea

[2]Center for Opto-Electronic Conversion System, Korea Institute of Science and Technology, Seoul 136-791, Korea

[*] *Corresponding author.* Tel: +82-2-958-5725. Email: ymjhon@kist.re.kr (Young Min Jhon)

◈Topological Description of SWNT-HL1 and SWNT-HL2

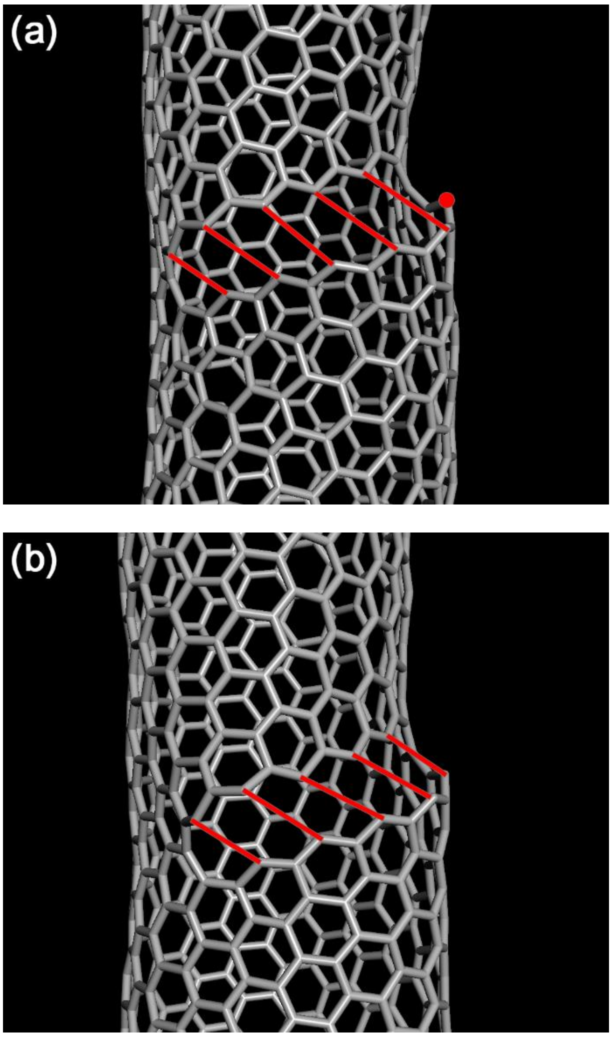

Figure S1. (a) The topologically-staggered structure (SWNT-HL1) and **(b)** the topologically-coherent structure (SWNT-HL2) of helical defects in SWNTs. A red circle indicates a lone connect point after the full topological pairing.

◈ Crack Length Determination in HL defects

Figure S2 shows the uncoiled graphene nanoribbon (the shaded object) of SWNTs for two-rotations of the helical growth. We assume that $\alpha = L_0/(\pi D)$ is constant, which means that the circumferential shape of HL defects is similar, independent of the diameter (D) and the larger chiral angle (θ) of SWNTs. In this regime, the crack-length (L) of HL defects can be determined by D, θ, and α using the following equation;

$$L = L_0/\cos\theta = \alpha\pi D/\cos\theta \propto D/\cos\theta$$

where α is a proportional constant that can be obtained from the structure of SWNT-HL1 sample.

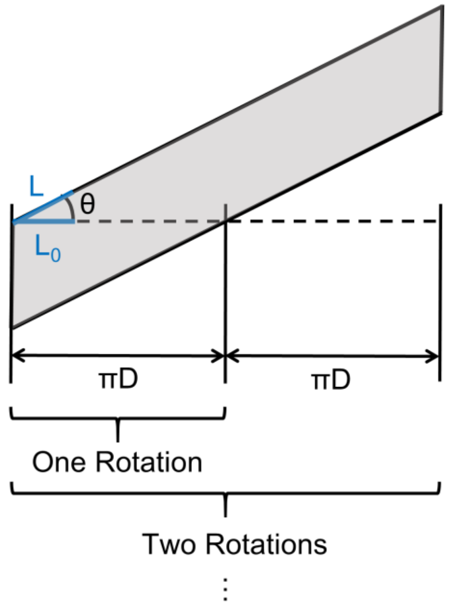

Figure S2. The diagram for crack length determination in HL defects.

◈ Tensile Stress-strain Curves and Fracture Structure of SWNT-HL1

(a)

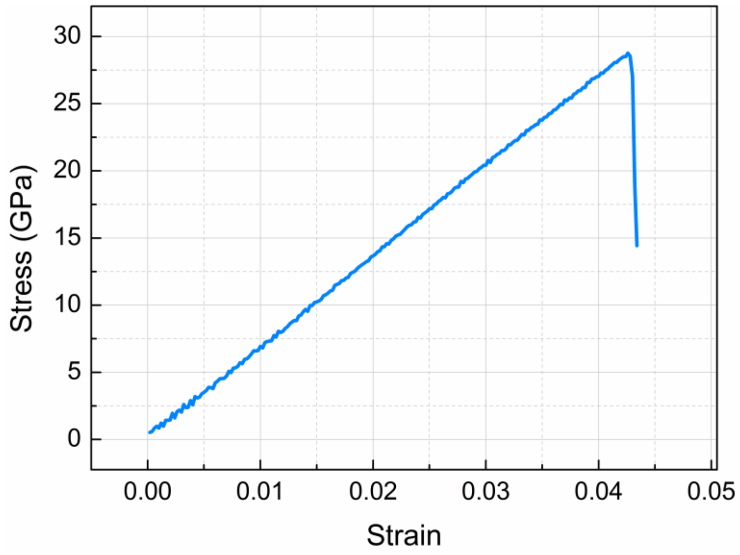

(b)

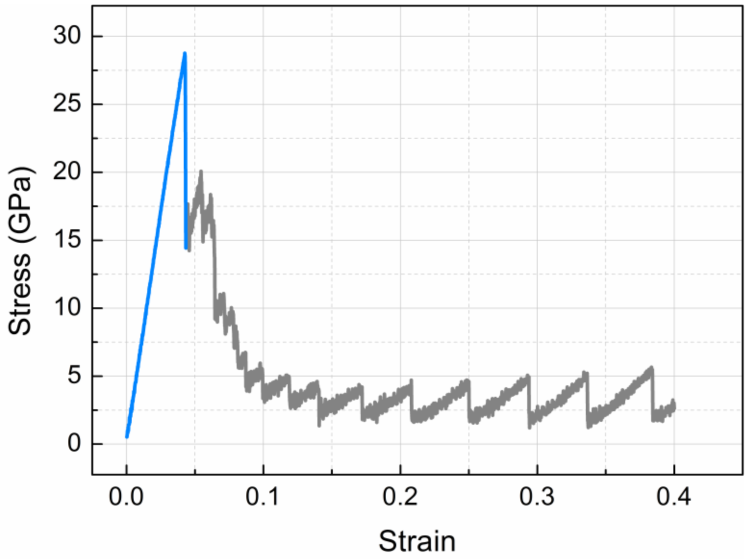

Figure S3. (a) Tensile stress-strain curve of SWNT-ND1 and **(b)** residual stresses (in gray) appearing during inhomogeneous deformation after tensile failure.

◈residual connection in SWNT-HL1 after Tensile Failure

◈Residual Connections after the Tensile Failure in SWNT-HL1

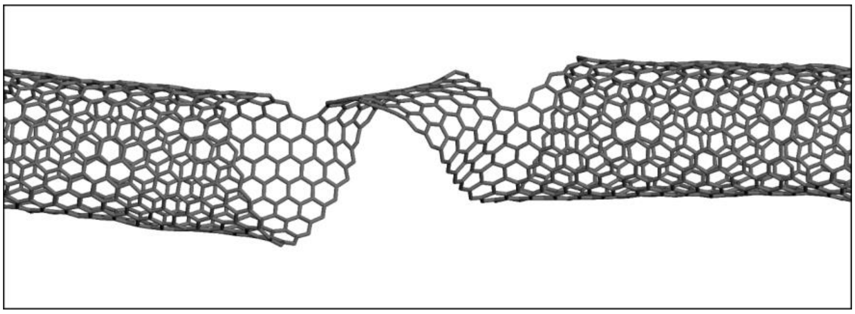

Figure S4. The typical structure of SWNT-HL1 appearing during inhomogeneous deformations after tensile failure. Some residual stresses occur after the tensile failure of SWNT-NL1, which is attributed to the connection via graphene nanoribbons that comes from unraveling of nodal structures.
